# Allogeneic ADSCs Induce the Production of Alloreactive Memory-CD8 T Cells through HLA-ABC Antigens

**DOI:** 10.3390/cells9051246

**Published:** 2020-05-18

**Authors:** Sung-Ho Chang, Hyun Je Kim, Chung-Gyu Park

**Affiliations:** 1 Departments of Oral Microbiology and Immunology, School of Dentistry and Dental Research Institute, Seoul National University, Seoul 03080, Korea; samuelsh@snu.ac.kr; 2 Department of Microbiology and Immunology, Seoul National University College of Medicine, Seoul 03080, Korea; hjkim0518@gmail.com; 3 Department of Dermatology, Samsung Medical Center, Seoul 06351, Korea; 4 Institute of Endemic Diseases, Medical Research center, Seoul National University College of Medicine, Seoul 03080, Korea; 5 Cancer Research Institute, Seoul National University College of Medicine, Seoul 03080, Korea; 6 Department of Biomedical Sciences, Seoul National University College of Medicine, Seoul 03080, Korea

**Keywords:** adipose-derived mesenchymal stem cells (ADSCs), direct pathway, alloreactive memory CD8 T cells, human leukocyte antigen (HLA), immunogenicity

## Abstract

We investigated the immunogenicity of allogeneic human adipose-derived mesenchymal stem cells (ADSCs) through the production of alloreactive-CD8 T and -memory CD8 T cells, based on their human leukocyte antigen (HLA) expression. In surface antigen analysis, ADSCs do not express co-stimulatory molecules, but expresses HLA-ABC, which is further increased by exposure to the pro-inflammatory cytokines as well as IFN-γ alone. For immunogenicity analysis, allogeneic ADSCs cultured in xenofree medium (XF-ADSCs) were incubated with the recipient immune cells for allogeneic–antigen stimulation. As a result, XF-ADSCs induced IFN-γ and IL-17A release by alloreactive-CD8 T cells and the production of alloreactive-CD8 T cell through a direct pathway, although they have immunomodulatory activity. In the analysis of alloreactive memory CD8 T cells, XF-ADSCs also significantly induced the production of CFSE-low-CD8 TEM and -CD8 TCM cells. However, HLA-blocking antibodies significantly inhibited the production of CFSE-low memory-CD8 T cells, indicating that HLAs are the main antigens responsible for the development of allogeneic ADSCs’ immunogenicity. These results suggested that HLA surface antigens expressed in allogeneic MSCs should be solved in order to address concerns related to the immunogenicity problem.

## 1. Introduction

Mesenchymal stem cells (MSCs), isolated originally from bone marrow aspirates, have been identified in most human tissues, including adipose tissue, umbilical cord blood, spleen, thymus and kidney [1,2,3,4,5]. As multipotent progenitor cells, MSCs possess the ability to differentiate into various cell types such as osteocytes, adipocytes, neural cells, vascular endothelial cells and pancreatic β-cells and have immunomodulation and self-renewal capability [6,7,8,9,10]. Thus, MSCs are attracting interest as a potential therapeutic agent for regenerative medicine, ocular, renal fibrosis, cardiac and immune disorders [11,12].

MSCs exhibit phenotypic and functional heterogeneity and are positive for CD13, CD44, CD73, CD90 and CD105 [13]. The cells do not express co-stimulatory molecules such as CD40, CD80 and CD86 [14]. In addition, major histocompatibility complex (MHC) class I molecules are expressed at low levels and MHC class II molecules are not expressed. These are expected to induce anergy in recipient T cells of allograft model [15,16]. Thus, the immunogenicity of allogeneic MSCs is considered to be low or absent [17,18]. On the other hand, there is concern about the immunogenicity of allogeneic MSCs because MSCs still partially express human leukocyte antigens (HLAs) that may be further increased when exposed to IFN-γ [19,20,21,22,23,24,25]. There are also reports that MSCs used to prevent allograft rejection rather promote inflammation or induce alloreactive cytotoxic T cell responses [23,26,27,28]. Thus, unlike traditional organ allografts, a better understanding of immunogenicity is currently needed to achieve the goals of safe- and effective-cell therapy using allogeneic MSCs [29,30,31,32,33].

T cells play an important role in allograft rejection through the recognition of allogeneic surface antigens [34,35]. T cells recognize allogeneic antigens via direct and indirect pathways [36]. The direct pathway is usually associated with acute allograft rejection and the indirect pathway is associated with chronic allograft rejection [24,37]. In the direct pathway, antigens are loaded on the donor antigen presenting cells (APCs). Meanwhile, antigens are loaded on the recipient APCs in the indirect pathway. Thus, HLAs recognized by the recipient T cells are key mediators in allograft rejection. In addition, memory T cells respond more rapidly to antigens than naïve T cells, as memory T cells are less dependent or independent on co-stimulation by CD80-CD28 and CD40-CD154 [38,39,40]. Alloreactive memory T cells can be generated from memory T cells that have previously been produced in response to infection, blood transfusion or pregnancy. However, alloreactive memory T cells are resistant to immunosuppressive agents, despite several recent advances [39,40,41,42]. In the phenotype of memory T cells, central memory T cells (TCM) are CD45RA^low^CD62L^hi^CCR7^hi^ and are located in lymph nodes, spleen and blood. Effector memory T cells (TEM) are CD45RA^low^CD62L^low^CCR7^low^ and are located in the spleen, blood and liver [43,44,45]. Both TCM and TEM contribute effectively to rejection [46,47,48,49].

It is important to first anticipate the immunogenicity of MSCs to improve the treatment efficiency and safe use of MSCs. For this study, surface markers that may be associated with the development of immunogenicity of ADSCs were investigated. In the immunogenicity evaluation, we examined the antigen recognition pathway of the recipient T cells to allogeneic XF-ADSCs. We also examined whether ADSCs induce immunogenicity such as the development of alloreactive memory-CD8 T cells in an ex vivo human models [29]. In this study, all allogeneic immune responses were performed in xenofree media containing autologous serum instead of FBS to eliminate the concern of the immune response by xenogeneic molecules [50,51,52].

## 2. Materials and Methods

### 2.1. Study Approval

Human ADSCs and adipose tissue, obtained with patient consent, were supplied by Prof. Oh IH and Rhie JW in accordance with institutional review board (IRB)-approved procedures of the Catholic University in Seoul, Korea. This study was approved by the IRB of the Hospital Biomedical Research Institute of Seoul National University (document number, 1403-036-563). All mouse studies were approved by the Institutional Animal Care and Use Committee of Seoul National University (document number, SNU-150310-2-2).

### 2.2. Preparation and Phenotypic Analysis of Human ADSCs

ADSCs from human females between 36 and 53 years of age were isolated from adipose tissue of the abdomen or breast (10 patients). Briefly, adipose tissue was finely cut with scissors and separated into adipocytes through digestion for 1 h in a water bath of 37 °C in Dulbecco’s phosphate-buffered saline (DPBS, Life Technologies, Grand Island, NY, USA) containing 0.1% collagenase type I (Life Technologies, Grand Island, NY, USA) [53]. The isolated adipocytes were immediately cultured in Dulbecco’s modified Eagle’s medium (DMEM, Life Technologies) with 10% fetal bovine serum (FBS, Life Technologies) (MSC-qualified, Life Technologies), 1% penicillin–streptomycin (Life Technologies) and 1% GlutaMAX™ (Life Technologies) for one day in a *T*-75 flask (Thermo Fisher, Carlsbad, CA, USA), and floating cells were removed by replacing the medium the next day. Periodically, the medium was exchanged to remove floating cells and the obtained ADSCs were used after specimen verification for mycoplasma contamination. For allogeneic–antigen stimulation (AAS), the isolated adipocytes were cultured in CellGenix MSC, a serum-free medium for MSCs (CellGenix, Portsmouth, NH, USA, 24803-0500), which is referred to as xenofree media in this study, without animal-derived components. The flask used here was coated with CELLstart humanized substrate (Life Technologies, A1014201). To analyze positive markers of ADSCs, cells were stained with the following monoclonal antibodies (mAbs): anti-human CD13 (BioLegend, San Diego, CA, USA), anti-human CD44, anti-human CD73, anti-human CD90, anti-human CD105 (eBioscience, San Diego, CA, USA). The following antibodies were used for surface antigen and co-stimulatory molecule analysis: anti-human CD80, anti-human CD86 (eBioscience), anti-HLA-ABC and anti-HLA-DR (BioLegend). To analyze anti-human natural killer group 2D ligand (NKG2DL), cells were stained with the following monoclonal antibodies (mAbs): anti-human MIC-A, anti-human MIC-B, anti-human ULBP1 and anti-human ULBP2/5/6 (R&D Systems, Minneapolis, MN, USA) (Table 1). To analyze changes in surface antigens on ADSCs, cells were stimulated with various recombinant human cytokines (interferon gamma (IFN-γ; 50 ng/mL, 34-8319-85), tumor necrosis factor alpha (TNF-α;10 ng/mL, 34-8329-85), interleukin (IL)-17A/F (50 ng/mL, 34-8178-85) and IL-23 (10 ng/mL, 14-8239-63; eBioscience) or their combinations. ADSCs that had been passaged two to eight times were used for phenotypic analysis using a FACS Canto II flow cytometer (BD Biosciences, San Diego, CA, USA). ADSCs were stained with these antibodies for 15 min at ice, washed with DPBS and analyzed by FACS or after fixing.

### 2.3. Analysis of Immunosuppressive Activity by ADSCs in Stimulated Mouse T Cells

Female C57BL/6 mice (7 weeks old) were purchased from Orient-Bio (Seongnam, Korea). Mouse CD3 T cells were isolated from their spleens using a mouse Pan T cell isolation kit (Miltenyi Biotec, Auburn, CA, USA) according to the manufacturer’s protocol. Separated CD3 T cells were labeled with 0.5 μM carboxyfluorescein succinimidyl ester (CFSE; Sigma-Aldrich, St Louis, MO, USA); subsequently, CFSE-labeled CD3 T cells were stimulated in a 24-well plate (Thermo Fisher) for 4 days in the presence of plate-coated anti-mouse CD3 (5 μg/mL) plus soluble anti-mouse CD28 (5 μg/mL) antibodies (eBioscience). CFSE-labeled CD3 T cells were suspended at 1 × 10^5^ cells/well in the plate. The cells were cultured in DMEM with 1 ng/mL IL-2 (eBioscience), 10% FBS, 1% penicillin–streptomycin and 1% GlutaMAX™. To examine immunosuppressive activity, human ADSCs were added at 7 × 10^4^ cells/well on the day of T cell stimulation or one day later. Proliferation of CFSE-labeled CD3 T cells was analyzed using flow cytometry and data were analyzed using FlowJo V 7.6.5 software (Tree Star, Ashland, OR, USA).

### 2.4. Preparation of T Cells and Td-PBMCs for Allogeneic–antigen Stimulation

Peripheral blood mononuclear cells (PBMCs) were isolated from blood donated by 5 healthy male and 5 healthy females between 26 and 46 years of age. CD3 T cells were isolated from PBMCs using a human Pan T cell isolation kit (Miltenyi Biotec) according to the manufacturer’s protocol. CD8 T cells were isolated from PBMCs using CD8 T isolation kits (Miltenyi Biotec). T cells separated for allogeneic–antigen stimulation were labeled with 0.5 μM CFSE for 5 min at room temperature and washed four times with xenofree media or DPBS. T cell-depleted PBMCs (Td-PBMCs) were isolated from PBMCs using a CD3 *T*-positive isolation kit (Miltenyi Biotec). For allogeneic–antigen stimulation, separated Td-PBMCs were irradiated at 10 Gy (IBL-437C, CIS Bio International, Gif-sur-Yvette, France) for their role as antigen presentation cells of B cells, as the function of B cells was significantly reduced at 20 Gy [54,55]. To examine the production of memory T cells, T cells were stained with anti-human CD3, anti-human CD4, anti-human CD8, anti-human CD45RA and anti-human CD62 L (BioLegend) antibodies, as well as with the respective isotype antibodies corresponding to these target antibodies. The phenotypic changes in T cells after allogeneic–antigen stimulation was analyzed by flow cytometry (BD Biosciences).

### 2.5. Allogeneic–Antigen Stimulation

The isolated ADSCs were maintained in xenofree media for MSCs (XF-ADSCs), and cells that had undergone one or two passages were used for allogeneic–antigen stimulation experiments. Four days before allogenic-antigen stimulation, XF-ADSCs were stimulated or not with the following recombinant human cytokines: IFN-γ and a combination of IFN-γ, IL-17A/F and IL-23 (eBioscience). Allogeneic XF-ADSCs were incubated with CD3 T cells and Td-PBMCs for allogeneic–antigen stimulation for 21 days. Specifically, for allogeneic–antigen stimulation via the indirect pathway, the detached XF-ADSCs were disrupted by repetitive thawing and freezing (five times) using liquid nitrogen; the disrupted XF-ADSCs were then added, at a volume ratio of 1 × 10^3^ cells/well, to a 12-well plate containing T cells and Td-PBMCs. The disrupted cells were added at days 0, 7 and 14 and stored at −20 °C until the experiment. To perform allogeneic–antigen stimulation via the direct pathway, XF-ADSCs were seeded at 1 × 10^3^ cells/well on a 12-well plate one day before the experiment. T cells and Td-PBMCs were seeded at 2.3 × 10^5^ cells/well and 1.3 × 10^5^ cells/well, respectively, in a 12-well plate on which XF-ADSCs were also plated. Td-PBMCs were added at days 0, 7 and 14 of these experiments. XF-ADSCs at 1 × 10^3^ cells/well were further added on day 7 for direct pathway experiments. Allogeneic–antigen stimulation was performed in xenofree media with 5% autologous serum from a blood donor, 1 ng/mL IL-2 (eBioscience), 1% penicillin–streptomycin and 1% GlutaMAX™. Half of the medium removed and replaced with fresh medium every 7 days.

### 2.6. Enzyme-Linked ImmunoSpot (ELISPOT) Analysis

Pro-inflammatory cytokine secretion from T cells in response to allogeneic–antigen stimulation was measured using an ELISPOT assay. Briefly, captured anti-human IFN-γ (BD Biosciences, San Diego, CA, USA) and anti-human IL-17A antibodies (Mabtech, West Street, OH, USA) were coated overnight at 4 °C on a 96-well filter plate (Millipore, Billerica, MA, USA, S2EM004M99) according to the manufacturer’s instructions. On day 14 of allogeneic–antigen stimulation, whole cells were harvested and washed and then evenly plated in four on 96-well filter plates using the same medium as that used for allogeneic–antigen stimulation. Ten percent of the cells harvested after allogeneic–antigen stimulation were used for flow cytometry analysis of CFSE-low T cell counts. The plates were further cultured for 3 days at 37 °C in 5% CO_2_. After incubation, the cells were removed from the plates and a biotin-conjugated detection antibody was added, followed by streptavidin-alkaline phosphatase, according to the manufacturer’s instructions (Mabtech). The plates were developed using a chromogenic substrate, nitro-blue tetrazolium and 5-bromo-4-chloro-3’-indolyphosphate (NBT/BCIP; Sigma-Aldrich). The number of spots obtained via the ELISPOT assay was calculated by dividing by the number of CFSE-low T cells. The number of spots was determined using an ELISPOT reader (AID GmbH, Strasbourg, Germany).

### 2.7. Treatment with Blocking- and Neutralizing-Antibodies

To neutralize cytokines, anti-human IFN-γ (eBioscience, 14-7318-85) and anti-human IL-17A antibodies (eBioscience, 16-7178-85) were used for allogeneic–antigen stimulation. Anti-HLA-ABC (BioLegend, 311412), anti-HLA-DR (BioLegend, 307612) and anti-HLA-DQ antibodies (BioLegend, 361502) were used to inhibit HLAs. Antibodies were added at 1 μg/mL every 7 days when the culture medium was changed.

### 2.8. Statistics

Student’s *t*-tests were used to compare the results between the two experimental groups. Statistically significant differences between multiple groups were tested using one-way analysis of variance (ANOVA) with the Kruskal–Wallis test and Dunn’s multiple comparison test. All results were generated using GraphPad Prism, version IV (Graph-Pad Software, Inc., San Diego, CA). All data are expressed as the mean ± SEM and the asterisks indicate significant differences from the control group (*, *p* < 0.05; **, *p* < 0.01; ***, *p* < 0.001).

## 3. Results

### 3.1. IFN-γ or Combined Cytokines Increased HLA-ABC Expression on the Surface of ADSCs, but not the Expression of Co-Stimulatory Molecules or NKG2DL

**Figure 1:**
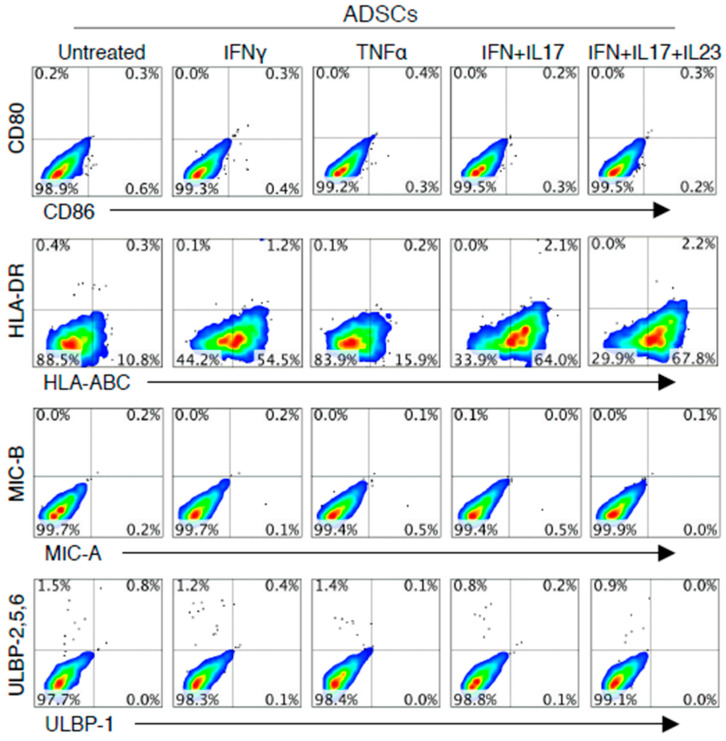
Effect of pro-inflammatory cytokines on the expression of human leukocyte antigens (HLAs) and co-stimulatory molecules on the surface of ADSCs. ADSCs were isolated from human adipose tissue and cultured in complete Dulbecco’s modified Eagle’s medium (DMEM). ADSCs that had been passaged fewer than eight times were used. ADSCs were stained with monoclonal antibodies (mAbs) against CD80 and CD86. ADSCs were additionally stained with mAbs against HLA-DR and HLA-ABC and for markers of NKG2DL with mAbs against MIC-A, MIC-B, ULBP1 and ULBP2/5/6. The data are representative of at least three experiments. IFN+IL-17: combination of IFN-γ and IL-17A/F; IFN+IL-17+IL-23: combination of IFN-γ, IL-17A/F and IL-23.

This study investigated the expression of human ADSCs surface markers, co-stimulatory molecules, HLAs and NKG2DL. ADSCs used in this experiment share positive markers of MSCs such as CD13, CD44, CD73, CD90 and CD105. As shown in Figure 1, ADSCs do not express CD80 and CD86 under both noninflammatory and inflammatory conditions. However, HLA-ABC expression of ADSCs was not only expressed in untreated ADSC but was further increased in the combination of IFN-γ, IL-17A/F and IL-23.

### 3.2. ADSCs Reduce the Proliferation of Anti-CD3- and Anti-CD28-Stimulated Mouse CD8 T Cells

**Figure 2:**
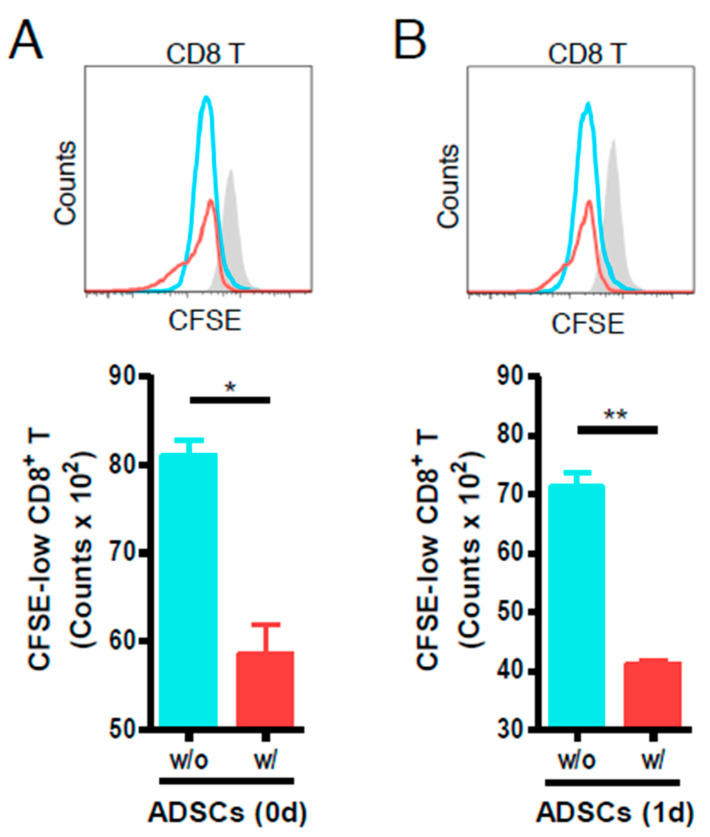
Immunosuppressive effects of human ADSCs on the proliferation of artificially stimulated mouse T cells. Mouse CD3 T cells were stimulated with plastic-coated anti-CD3 and soluble anti-CD28 antibodies in a 24-well plate. (**A**) CD3 T cells were suspended (at 1 × 10^5^ cells/well) with ADSCs (at 7 × 10^4^ cells/well) on the day of the stimulation (*n* = 4 (w/o), *n* = 5 (w/)). (**B**) CD3 T cells were suspended at 1 × 10^5^ cells/well on the day of the stimulation and ADSCs were added at 7 × 10^4^ cells/well on the day after stimulation of T cells (*n* = 4 (for each sample)). Carboxyfluorescein succinimidyl ester (CFSE)-low-CD8 T cells were analyzed by flow cytometry on day 4 after stimulation. T cells cultured with ADSCs are red line and T cells without ADSCs are sky blue line. CFSE-labeled T cells that are not artificially stimulated are gray-filled histograms; *, *p* < 0.05; **, *p* < 0.01.

As shown in Figure 2, ADSCs added on the day of stimulation, although not of a dramatic immunosuppressive effect, reduced the number of proliferated CD8 T cells as compared to the control without ADSCs (Figure 2A). In addition, ADSCs reduced T cell proliferation, even when they were added one day after T cell stimulation (Figure 2B). These results indicated that human ADSCs exert immunosuppressive effects during the proliferation of artificially stimulated T cells.

### 3.3. XF-ADSCs Induce IFN-γ and IL-17A Release by Alloreactive CD3 T Cells in Allogeneic–antigen Stimulation Primarily Via the Direct Pathway

**Figure 3:**
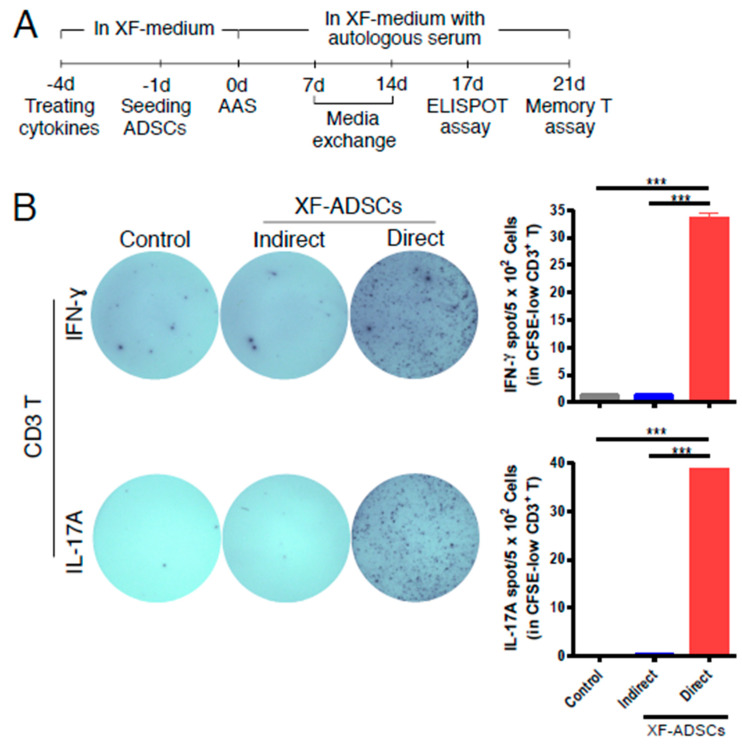
Analysis of antigen recognition pathways for immunogenicity evaluation of XF-ADSCs via allogeneic–antigen stimulation. (**A**) A three-week experimental scheme is depicted. (**B**) The antigen recognition pathway of CD3 T cells toward XF-ADSCs was determined by analysis of indirect and direct pathways in allogeneic–antigen stimulation, as described in ELISPOT analysis of the Methods. For allogeneic–antigen stimulation, CFSE-labeled CD3 T cells and T cell-depleted peripheral blood mononuclear cells (Td-PBMCs) were cultured with XF-ADSCs or disrupted XF-ADSCs. For indirect pathway analysis, the disrupted XF-ADSCs were added on the first day of this response and on days 7 and 14 (*n* = 4 (for each sample)). For direct pathway analysis, XF-ADSCs were seeded on a 12-well plate the day before allogeneic–antigen stimulation and were then added additionally for sensitization after 7 days of this response. Spots of IFN-γ or IL-17A secreted by CD3 T cells were analyzed by ELISPOT. The control does not contain XF-ADSCs and consist only of CD3 T cells and Td-PBMCs from healthy donor. The data were repeated at least three times; ***, *p* < 0.001. AAS: allogeneic–antigen stimulation.

As shown in Figure 3B, XF-ADSCs (xenofree medium-cultured ADSCs) significantly induced IFN-γ and IL-17A release by CFSE-low CD3 T cells through the direct pathway rather than indirect pathway.

### 3.4. XF-ADSCs Elicit Significant Production of CFSE-Low CD8 T Cells in Allogeneic–Antigen Stimulation

**Figure 4:**
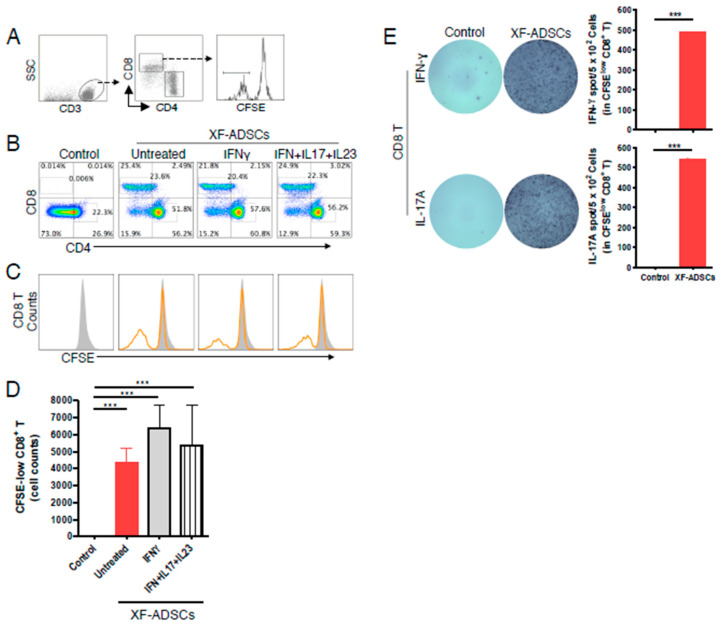
Production of alloreactive CD8 T cells during allogeneic–antigen stimulation. XF-ADSCs were cultured with CD3 T cells and Td-PBMCs via the direct pathway, after 3 weeks whole cells were harvested and CD8 T cells were analyzed by flow cytometry. (**A**) A scheme for the flow cytometry analysis is shown. (**B**) The dot plots indicated the distribution of CD4 T and CD8 T cells on day 21 after allogeneic–antigen stimulation compared with the control (*n* = 9 (for each sample)). (**C**) The histogram showed the CFSE-low-CD8 T cells in the CD8 T cell distribution of (b). (**D**) Graphical representation of the number of CFSE-low CD8 T cells corresponding to (C) (*n* = 16 (control), *n* = 14 (for each sample)). (**E**) For ELISPOT analysis, XF-ADSCs were cultured with CD8 T cells and Td-PBMCs via the direct pathway. After 2 weeks, whole cells were harvested and plated in quadruplicate on a 96-well filter plate (*n* = 4 (for each sample)). Spots of IFN-γ or IL-17A secreted by CD8 T cells were analyzed by ELISPOT. The controls do not contain XF-ADSCs and consist only of CD3 T cells (or CD8 T) and Td-PBMCs from healthy donor. The data were repeated at least three times; ***, *p* < 0.001.

As shown in Figure 4B, the ratio of CD8 T cells incubated with XF-ADSCs or pretreated XF-ADSCs was significantly increased compared with that in the control not containing XF-ADSCs. Here, most of the CD8 T cells in the control did not survive until 3 weeks because there was no antigen stimulation. In addition, we analyzed the production of CFSE-low CD8 T cells to more accurately elucidate the production of alloreactive CD8 T cells. As a result, histogram analysis showed that CFSE-low CD8 T cells toward XF-ADSCs were significantly increased compared with that in the control (Figure 4C,D). In addition, XF-ADSCs significantly induced IFN-γ and IL-17A release by alloreactive CD8 T cells compared with that in the control (Figure 4E).

### 3.5. XF-ADSCs Cause Significant Production of CFSE-Low Memory-CD8 T Cells in Allogeneic–antigen Stimulation

**Figure 5:**
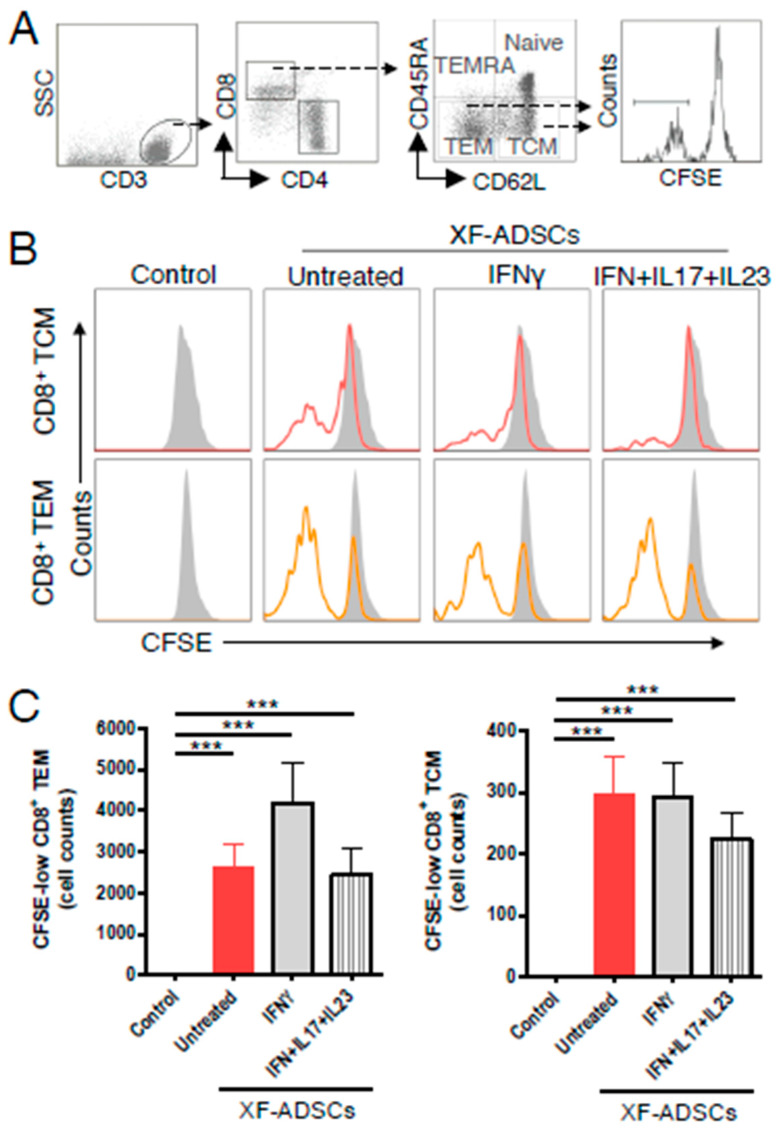
Production of alloreactive memory-CD8 T cells during allogeneic–antigen stimulation. XF-ADSCs were cultured with CD3 T cells and Td-PBMCs via the direct pathway, after 3 weeks whole cells were harvested and CD8 T cells were analyzed by flow cytometry. (**A**) Scheme for the flow cytometry analysis. (**B**) The histogram showed the CFSE-low memory-CD8 T cells in the population of memory-CD8 T cells on the 21st day after allogeneic–antigen stimulation, compared with the control. (**C**) Graphical representation of the number of CFSE-low memory-CD8 T cells corresponding to (B) (*n* = 15 (each control and untreated), *n* = 13 (each IFNγ and IFN+IL17+IL23)). The control does not contain XF-ADSCs and consist only of CD3 T cells and Td-PBMCs from healthy donor. The data were repeated at least three times; ***, *p* < 0.001.

As shown in Figure 5B,C, XF-ADSCs significantly induced the production of CFSE-low-CD8 TEM and -CD8 TCM cells compared with that in the control. It also means that XF-ADSCs do not adequately suppress the production of alloreactive memory T cells.

### 3.6. HLA-Blocking Antibodies Inhibit the Production of Alloreactive Memory-CD8 T Cell in Allogeneic–Antigen Stimulation

**Figure 6:**
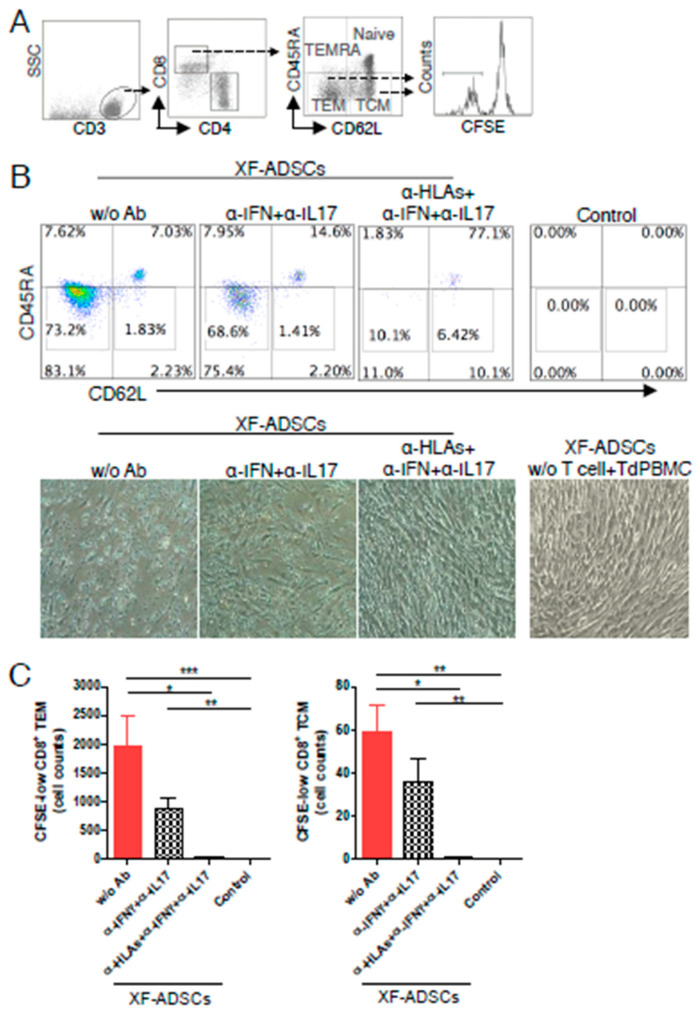
HLA-blocking antibodies inhibit the production of alloreactive memory-CD8 T cells during allogeneic–antigen stimulation. XF-ADSCs were cultured with CD3 T cells and Td-PBMCs via the direct pathway, after 3 weeks whole cells were harvested and memory-CD8 T cells were analyzed by flow cytometry. (**A**) Scheme for the flow cytometry analysis. (**B**) CD3 T cells were cultured for 3 weeks with or without neutralizing or blocking antibodies during allogeneic–antigen stimulation. A combination of anti-human IL-17A and anti-human IFN-γ antibodies was used to neutralize pro-inflammatory cytokines. A combination of anti-HLA-ABC, anti-HLA-DR and anti-HLA-DQ antibodies was used to block HLAs. The dot plots showed the population of memory-CD8 T cells on the 21st day after allogeneic–antigen stimulation, compared with the control. (**C**) Graphical representation of the number of CFSE-low memory-CD8 T cells corresponding to (B) (*n* = 7 (each CD8 TEM), *n* = 6 (each CD8 TCM)). The control does not contain XF-ADSCs and consist only of CD3 T cells and Td-PBMCs from healthy donor. The data were repeated at least three times; *, *p* < 0.05; **, *p* < 0.01; ***, *p* < 0.001. α-IFN+α-IL17: combination of anti-human IFN-γ and anti-human IL-17A antibodies; α-HLAs: combination of anti-HLA-ABC, HLA-DR and anti-HLA-DQ antibodies.

As shown in Figure 6B,C, addition of HLA-blocking antibodies inhibited the production of CFSE-low memory-CD8 T cells significantly compared with that in the XF-ADSCs without antibodies. However, the addition of the neutralizing antibody showed only a slight inhibitory effect. Here, most of the CD8 T cells in the control without allogeneic ADSCs did not survive until 3 weeks. These results indicated that the HLA-ABC surface antigens on allogeneic ADSCs are a major contributor to the production of alloreactive memory-CD8 T cells.

## 4. Discussion

MSCs are attracting attention because of its immunosuppression and differentiation potential in immunotherapy and regenerative medicine. However, there is also concern about the immunogenicity associated with MSC allografts [50,56,57,58]. The aim of this study was therefore to determine whether ADSCs induce immunogenicity as alloreactive T cells in an ex vivo human allograft model, which is referred to as allogeneic–antigen stimulation in this study. Then we examined what the major antigens of this immunogenicity are. Thus, we first investigated the surface markers of ADSCs that may be involved in the activation of T and NK cells, which play an important role in allograft rejection. Previous reports have shown that IFN-γ increases the expression of HLA-ABC and induces the expression of HLA-DR in MSCs [24,59]. NKG2D binding to NKG2DL is also expressed in NK cells and CD8 T cells, and expression of this ligand in ADSCs may be associated with allograft rejection [60,61,62]. Thus, we investigated the expression levels of HLA-ABC, HLA-DR, co-stimulatory molecules and NKG2DL on the surface of ADSCs in noninflammatory and inflammatory environments. In addition, the combination of IFN-γ, IL-17A/F and IL-23, referred to in the present study as cytokine combination, was designed for a disease model such as inflammatory bowel disease (IBD) or experimental autoimmune encephalomyelitis (EAE) [63]. Similar to previous reports, our experiments showed that ADSCs do not express co-stimulatory molecules and NKG2DL and expresses HLA-ABC. However, HLA-ABC expression is further increased in various inflammatory conditions and HLA-DR is also very weakly expressed (Figure 1). However, it should be noted that the level of HLAs and NKG2DL expression at the MSC surface may vary depending on tissue origin [12,64]. These results indicated that HLA-ABC expression on ADSCs is sensitive to pro-inflammatory cytokines, particularly IFN-γ, compared with that elicited by untreated ADSCs. Thus, these results suggested that the immunogenicity studies on allogeneic ADSCs need to be examined in association with the expression of HLA-ABC.

Before evaluating the immunogenicity, we examined the inhibitory effect of ADSCs to regulate the inflammatory response. We have shown that human ADSCs could reduce the proliferation of artificially stimulated mouse CD8 T cells (Figure 2). This was not a dramatic effect, but it is consistent with the previous reports that they can partially suppress lymphocyte proliferation in vitro [65,66]. Based on this, we investigated whether allogeneic ADSCs exhibit immunogenicity during longer-term reaction with human immune cells.

This study investigated whether human ADSCs cause release of the pro-inflammatory cytokines by alloreactive T cells in the long-term allogeneic immune response, referred to in the present study as allogeneic–antigen stimulation. Immunogenicity assays of XF-ADSCs were performed in a xenofree media with an autologous serum in an allogeneic–antigen stimulation experiment. As a result, XF-ADSCs induced IFN-γ and IL-17A release by CFSE-low-CD3 T cells primarily via direct pathway rather than in an indirect manner (Figure 3B). Although B cells were used as antigen presenting cells in this study, immunogenicity analysis through an indirect pathway may reduce the immune response, but there is no doubt that the immunogenicity of ADSCs is significantly induced by direct pathway. There is also a previous report that antigen recognition by the direct pathway in allograft is 100 times higher than that of the indirect pathway [67]. These results suggested that allogeneic ADSCs can induce antigen-specific activity of recipient T cells. In addition, these results suggested that allogeneic ADSCs exhibit immunogenicity through the long-term allogeneic–antigen stimulation although it does not express the co-stimulatory molecules and has immunosuppressive effects.

Regarding MHC restriction, CD4 T cells can recognize MHC Class II and CD8 T cells can recognize MHC class I [22,36,68]. Thus, immunogenicity toward XF-ADSCs was further evaluated by analyzing the production of alloreactive CD8 T cells after long-term allogeneic–antigen stimulation. As a result, XF-ADSCs induced the production of CFSE-low CD8 T cells significantly through the direct pathway (Figure 4B–D). In addition, XF-ADSCs induce IFN-γ and IL-17A release by CFSE-low-CD8 T cells through direct pathway (Figure 4E). However, XF-ADSCs pretreated with a combination of pro-inflammatory cytokines did not induce an additional increase of CFSE-low CD8 T cells compared with that in untreated XF-ADSC. In the previous study, cytotoxicity of alloreactive CD8 T toward ADSCs with significantly increased HLA-ABC was faster than untreated ADSCs [28]. Thus, these results suggested that pre-treated ADSCs may be associated with a faster onset time than the increase of alloreactive CD8 T cells in an *ex vivo*. The production of CFSE-low CD4 T cells toward XF-ADSCs was also induced, but no significant increase was observed (data not shown). This may be associated with a very low level of HLA-DR expressed in ADSCs. Overall, these results indicated that XF-ADSCs can induce IFN-γ and IL-17A release of alloreactive CD8 T cells and the production of alloreactive CD8 T cells through direct pathway, which may be associated with HLA-ABC expression.

Alloreactive memory T cells represent a challenge to successful allograft transplantation because effective inhibitors of these cells are not available [39,41,42,69]. In addition, the production of alloreactive memory T cells is important because the cells are less dependent on co-stimulatory molecules or are independent [38,39]. Thus, we investigated whether XF-ADSCs induce the production of alloreactive memory CD8 T cells in the long-term allogeneic–antigen stimulation. As a result, both untreated XF-ADSCs and pretreated XF-ADSCs increased the number of CFSE-low-CD8 TEM and -CD8 TCM cells significantly compared with that in the control, suggesting that alloreactive memory CD8 T cells were induced (Figure 5). In addition, these results showed that approximately 70% of alloreactive CD8 T cells are alloreactive memory CD8 T cells (Figure 4 and Figure 5). Our results that allogeneic human ADSCs induced alloreactive memory-CD8 T cells are also supported by previous reports demonstrating the production of memory T cells in response to allogeneic MSC using a mouse model [70]. These results demonstrated that the use of allogeneic ADSCs leads to the production of alloreactive CD8 TEM and CD8 TCM cells, which can occur independently of co-stimulatory molecules and is associated with important immunogenicity-related concerns. Taken together, the occurrence of these memory CD8 T cells implies that long-term survival of allogeneic ADSCs may be affected.

We investigated the main causes of production of alloreactive memory-CD8 T cells toward XF-ADSCs. In the above experiments, it was suggested that the production of alloreactive CD8 T cells toward ADSCs may be related to the expression of HLA-ABC surface antigens, since the T cells were activated through direct pathway in xenofree culture condition containing autologous serum. To further validate this, neutralizing antibodies toward pro-inflammatory cytokines and HLA-blocking antibodies toward MHC class I and II molecules were added during allogeneic–antigen stimulation through the direct pathway. As a result, the HLA-ABC blocking antibodies toward MHC class I molecules inhibited the production of CFSE-low-CD8 TEM and -CD8 TCM significantly compared with that in the control (Figure 6B,C). In addition, in morphology, XF-ADSCs incubated with HLA-blocking antibodies toward MHC class I showed more effective viability than that of XF-ADSCs without such antibodies, which is consistent with previous reports that studied cytotoxic effects toward allogeneic ADSC (Figure 6B) [28]. The results suggested that the production of alloreactive memory-CD8 T cells is associated with the expression of HLA-ABC on allogeneic ADSCs. The results are also supported by previous reports that MSCs can be rejected by mismatched-MHC in the allograft model [25,29,71]. Collectively, these results indicated that HLA-ABC expressed on the allogeneic ADSCs are a major contributor to the induction of immunogenicity, which is associated with the production of alloreactive-CD8 T and -memory-CD8 T cells.

## 5. Conclusions

This study showed that XF-ADSCs have immunomodulatory effects and do not express co-stimulatory molecules, but induced IFN-γ and IL-17A release by alloreactive CD8 T cells in long-term allogeneic–antigen stimulation through a direct pathway. Unfortunately, XF-ADSCs also induced the production of alloreactive-memory CD8 T cells. HLA-ABC blocking antibodies effectively inhibited the production of alloreactive memory-CD8 T cells toward XF-ADSCs, suggesting that HLA-ABC was a major cause of immunogenicity. Thus, these results indicated that allogeneic ADSCs that cause the production of alloreactive memory CD8 T cells may contribute not only to their own rejection, but also to the second-set rejection of other organs or cells. Therefore, these results suggested that HLA surface antigens expressed in allogeneic MSCs should be solved in order to address concerns related to the immunogenicity problems [29]. For this, developing strategies such as HLA matching, autologous MSCs or immunosuppressive use may be considered [25,72].

## Figures and Tables

**Table 1:** Antibodies to surface markers of allogeneic human adipose-derived mesenchymal stem cells (ADSCs) and information about them.

Antibody Target	Antibody Name (Clone)	Antibody Type	Manufacturer
ADSC-surface marker	CD13 (WM15)	Monoclonal	BioLegend
	CD44 (IM7)	Monoclonal	eBioscience
	CD73 (AD2)	Monoclonal	eBioscience
	CD90 (5E10)	Monoclonal	eBioscience
	CD105 (MJ7/18)	Monoclonal	eBioscience
Co-stimulatory molecule	CD80 (2D10.4)	Monoclonal	eBioscience
	CD86 (IT2.2)	Monoclonal	eBioscience
Human leukocyte antigen	HLA-ABC (W6/32)	Monoclonal	BioLegend
	HLA-DR (L243)	Monoclonal	BioLegend
NKG2DL	MIC-A (159227)	Monoclonal	R&D Systems
	MIC-B (236511)	Monoclonal	R&D Systems
	ULBP-1 (170818)	Monoclonal	R&D Systems
	ULBP-2/5/6 (165903)	Monoclonal	R&D Systems

## Abbreviations

XF-ADSCs	Allogeneic ADSCs cultured in xenofree medium
TCM	central memory T cells
TEM	effector memory T cells
Td-PBMCs	T cell-depleted PBMCs
AAS	allogeneic–antigen stimulation
DPBS	Dulbecco’s phosphate-buffered saline
PBMCs	peripheral blood mononuclear cells
